# Notation of Javanese *Gamelan* dataset for traditional music applications

**DOI:** 10.1016/j.dib.2024.110116

**Published:** 2024-02-06

**Authors:** Arik Kurniawati, Eko Mulyanto Yuniarno, Yoyon Kusnendar Suprapto, Noor Ifada, Nur Ikhsan Soewidiatmaka

**Affiliations:** a Department of Electrical Engineering, Institut Teknologi Sepuluh Nopember, Surabaya, Indonesia; b Department of Computer Engineering, Institut Teknologi Sepuluh Nopember, Surabaya, Indonesia; c Department of Informatics, University of Trunojoyo Madura, Bangkalan, Indonesia; d Soewidiatmaka Gamelan, Bandung, Indonesia

**Keywords:** Notation, Javanese *gamelan*, Music generation, Deep learning

## Abstract

The Javanese *gamelan* notation dataset comprises Javanese *gamelan gendhing* (song) notations for various *gamelan* instruments. This dataset includes 35 songs categorized into 7 song structures, which are similar to genres in modern music. Each song in this dataset includes the primary melody and notations for various instrument groups, including the *balungan* instruments group (*saron, demung,* and *slenthem*), the *bonang barung* and *bonang penerus* instruments, the *peking* instrument group, and the structural instruments group (*kenong, kethuk, kempyang, kempul,* and *gong*). The primary melody is derived from https://www.gamelanbvg.com/gendhing/index.php, a collection of Javanese *gamelan* songs. On the other hand, the notation of each instrument group is the result of our creation by following the rules of *gamelan* playing on each instrument. In Javanese *gamelan* songs, usually written only the main melody notation in the form of numerical notation and the characteristics of the song, such as song title, song structure type, rhythm, scale and mode of the song. Naturally, this is not an easy task for a beginner *gamelan* player, but a more complete notation will make it easier for anyone who wants to play *gamelan*. Each song is compiled into a sheet of music, which is presented in a Portable Document Format (PDF) file. This dataset is valuable for developing deep learning models to classify or recognize Javanese *gamelan* songs based on their instrument notations, as previous *gamelan* research has mostly used audio data. Furthermore, this dataset has the capability to automatically generate Javanese *gamelan* notation for songs of similar types. Additionally, it will be useful for educational purposes to facilitate the learning of Javanese *gamelan* songs and for the preservation of traditional Javanese *gamelan* music.

Specifications Table

**Table:** 

Subject	Arts and Humanities, Computer Science
Specific subject area	Music Classification, Music Recognition, and Music Generation
Data format	Raw
Type of data	PDF documents
Data collection	This dataset comprises 35 notation-based Javanese *gamelan* songs, organized into 7 song structures: *Sampak, Srepegan, Ayak-Ayakan, Lancaran, Bubaran, Ketawang,* and *Ladrang*. Each song structure contains 5 songs. The main melody is obtained from https://www.gamelanbvg.com/gendhing/index.php as a data source, and each song consists of notations of several *gamelan* instrument groups made by *gamelan* expert. The instrument groups comprise of *Balungan,* consisting of *Demung, Saron,* and *Slenthem, Peking, Bonang Barung* and *Bonang Penerus,* and *Structural Instruments,* consisting of *Kenong, Kethuk, Kempyang, Kempul, and Gong*.
Data source location	Department of Informatics Engineering, University of Trunojoyo Madura Bangkalan Jawa Timur 69162 Indonesia
Data accessibility	Repository name: Javanese *Gamelan* Notation Data identification number: 10.17632/dvrscpc6vc.1 Direct URL to data: https://data.mendeley.com/datasets/dvrscpc6vc/1
Related research article	A. Kurniawati, E. M. Yuniarno, Y. K. Suprapto, & A. N. I. Soewidiatmaka, Automatic note generator for Javanese gamelan music accompaniment using deep learning. International Journal of Advances in Intelligent Informatics, 9(2), 231-248 (2023). https://doi.org/10.26555/ijain.v9i2.1031[1].

## 1 Value of the Data

• This dataset provides comprehensive details on the complete notation of various Javanese *gamelan* instrument groups within a song that has been validated by *gamelan* expert. Naturally, Javanese *gamelan* songs only contain notations for the main melody and the characteristics of the song.
• This dataset will benefit a variety of communities, including researchers, novice *gamelan* players, and artists, by preserving traditional *gamelan* music.
• This dataset can also be used to build other similar song notation generators for the *balungan, bonang barung* and *bonang penerus, peking* and structural instrument groups.
• Furthermore, this dataset can also be used for the recognition and classification of Javanese *gamelan* songs based on their instrument notation.

## 2 Background

• The Javanese *gamelan* notation dataset includes notation for several instrumental groups. This makes it easier for beginners to learn *gamelan*. Furthermore, a complete notation helps to preserve and introduce Javanese *gamelan*.
• It can be used as a model for automatic *gamelan* song generators with similar types. Furthermore, the instrument notation can be useful for classification and recognition purposes.

## 3 Data Description

Javanese *gamelan* is an Indonesian art. In playing *gamelan* instruments, there are various rules that follow the playing pattern, which is better known as *karawitan*, while the instrument is called *gamelan* [2,3]. Javanese *gamelan* specifically refers to *gamelan* from Java, as there are other forms of *gamelan* found in different parts of Indonesia, including Balinese *gamelan*, Sundanese *gamelan*, Lombok, Madura, and more. The area from which the *gamelan* was developed indicates the style and pattern of their local tradition. The *gamelan* is commonly used for traditional art performances, religious ceremonies and other local traditions.

**Fig. 1:**
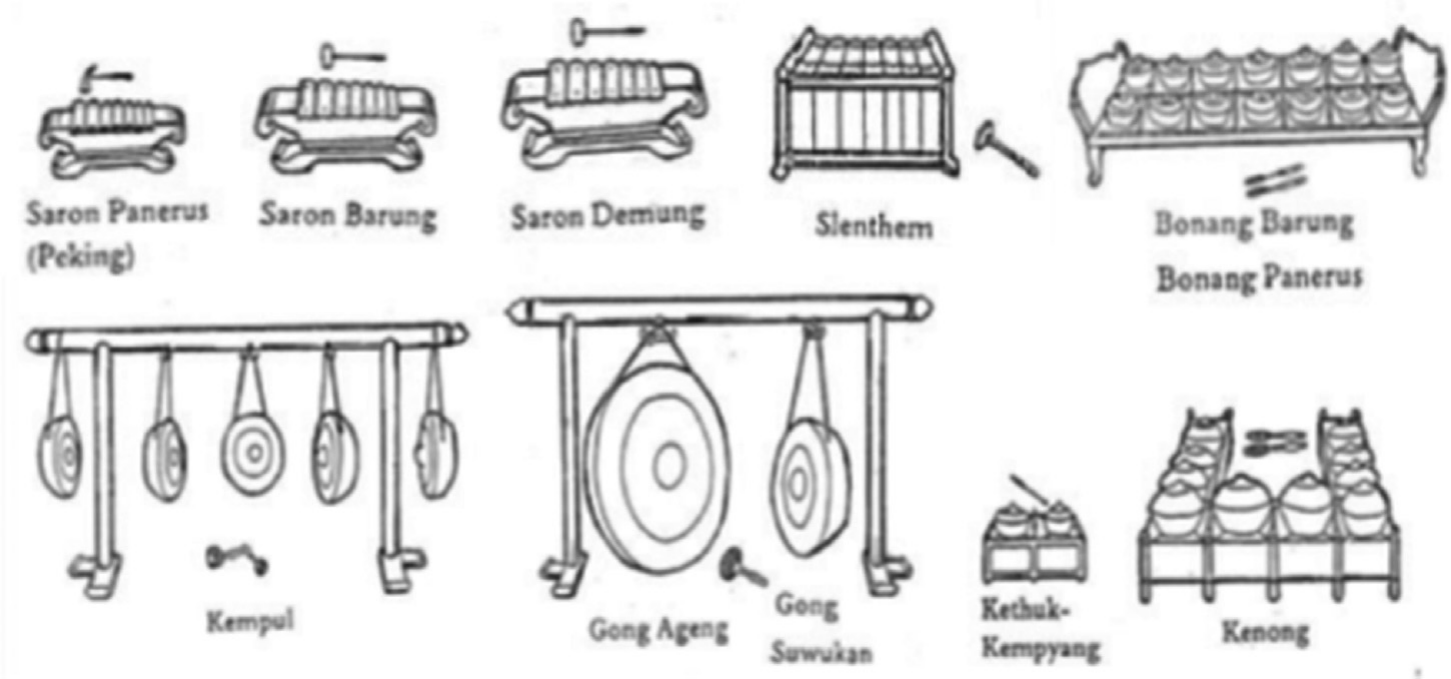
Instrument Javanese *gamelan*.

The Javanese *Gamelan* Notation dataset comprises 35 songs divided into 7 types of song structures, with each structure containing 5 songs. Each song is organized into files based on the basic melody of the song and the notation of several groups of instruments, such as: *balungan* instruments (i.e., *saron, demung,* and *slenthem*), *peking* instrument, *bonang barung* and *bonang penerus* instruments, and structural instruments (*kenong, kethuk, kempyang, kempul,* and *gong*). Fig. 1 illustrates the detailed instruments of the Javanese *gamelan* [4].

**Fig. 2:**
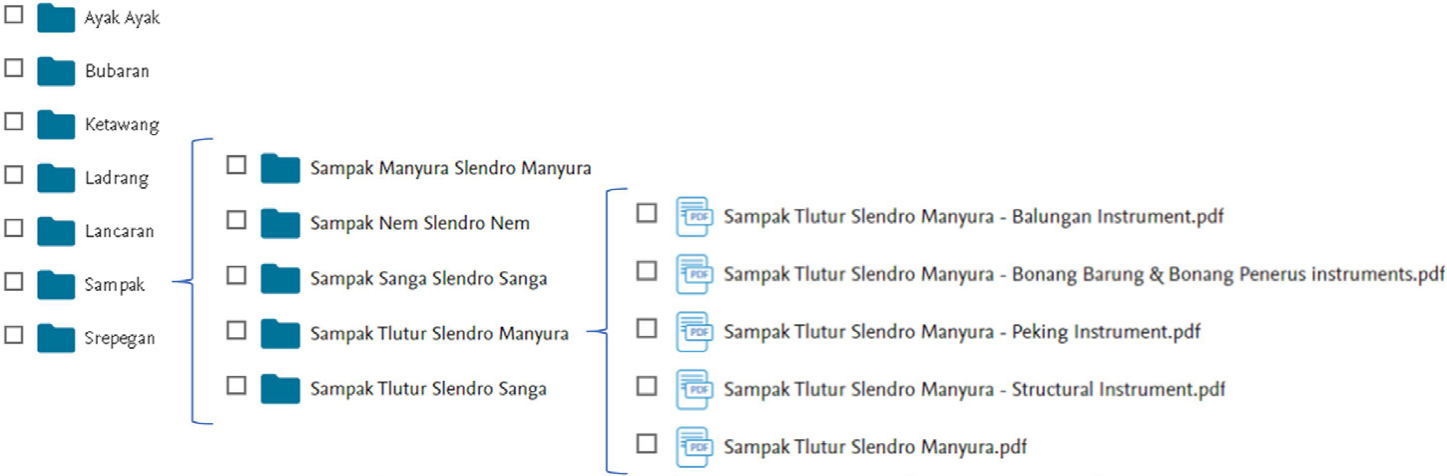
Organizing file of Javanese *gamelan* notation dataset.

The documents were organized in the following order: *Sampak, Srepegan, Ayak-Ayakan, Lancaran, Ketawang,* and *Bubaran* are folders grouped by song structure. Furthermore, each song structure contains 5 folders that indicate the name of the song. Each song folder includes a document with one file containing the song's main melody obtained from https://www.gamelanbvg.com/gendhing/index.php, and four files each containing the notation for the *balungan, bonang barung* and *bonang penerus, peking*, and structural instrument groups, as shown in Fig. 2. The notation of main melodies and instruments is presented using *gamelan* numeric symbols and is saved in a PDF file. The list of songs for this dataset provided in Table 1 below.

**Table 1:** List of songs in the Javanese *gamelan* dataset.

No	Song title	Song structure	Laras	Pathet
1.	*Sampak Manyura Laras Slendro Pathet Manyura*	*Sampak*	*Slendro*	*Manyura*
2.	*Sampak Nem Laras Slendro Pathet Nem*	*Sampak*	*Slendro*	*Nem*
3.	*Sampak Sanga Laras Slendro Pathet Sanga*	*Sampak*	*Slendro*	*Sanga*
4.	*Sampak Tlutur Laras Slendro Pathet Manyura*	*Sampak*	*Slendro*	*Manyura*
5.	*Sampak Tlutur Laras Slendro Pathet Sanga*	*Sampak*	*Slendro*	*Sanga*
6.	*Srepegan Manyura Laras Slendro Pathet Manyura*	*Srepegan*	*Slendro*	*Manyura*
7.	*Srepegan Nem Laras Slendro Pathet Nem*	*Srepegan*	*Slendro*	*Nem*
8.	*Srepegan Sanga Laras Slendro Pathet Sanga*	*Srepegan*	*Slendro*	*Sanga*
9.	*Srepegan Tlutur Laras Slendro Pathet Manyura*	*Srepegan*	*Slendro*	*Manyura*
10.	*Srepegan Tlutur Laras Slendro Sanga*	*Srepegan*	*Slendro*	*Sanga*
11.	*Ayak-Ayakan Manyura Laras Slendro Pathet Manyura*	*Ayak-Ayakan*	*Slendro*	*Manyura*
12.	*Ayak-Ayakan Nem Laras Slendro Pathet Nem*	*Ayak-Ayakan*	*Slendro*	*Nem*
13.	*Ayak-Ayakan Pamungkas Laras Slendro Pathet Manyura*	*Ayak-Ayakan*	*Slendro*	*Manyura*
14.	*Ayak-Ayakan Sanga Laras Slendro Pathet Sanga*	*Ayak-Ayakan*	*Slendro*	*Sanga*
15.	*Ayak-Ayakan Umbul Donga Laras Slendro Pathet Manyura*	*Ayak-Ayakan*	*Slendro*	*Manyura*
16.	*Lancaran Kuda Nyongklang Laras Pelog Pathet Barang*	*Lancaran*	*Pelog*	*Barang*
17.	*Lancaran Maesa Kurda Laras Slendro Pathet Sanga*	*Lancaran*	*Slendro*	*Sanga*
18.	*Lancaran Manyar Sewu Laras Slendro Pathet Manyura*	*Lancaran*	*Slendro*	*Manyura*
19.	*Lancaran Rena Rena Laras Slendro Pathet Manyura*	*Lancaran*	*Slendro*	*Manyura*
20.	*Lancaran Sarung Jagung Laras Pelog Pathet Barang*	*Lancaran*	*Pelog*	*Barang*
21.	*Bubaran Arum Arum Laras Pelog Pathet Barang*	*Bubaran*	*Pelog*	*Barang*
22.	*Bubaran Kembang Pacar Laras Pelog Pathet Nem*	*Bubaran*	*Pelog*	*Nem*
23.	*Bubaran Purwaka Laras Pelog Pathet Nem*	*Bubaran*	*Pelog*	*Nem*
24.	*Bubaran Sembunggilang Laras Slendro Pathet Sanga*	*Bubaran*	*Slendro*	*Sanga*
25.	*Bubaran Udan Mas Laras Pelog Pathet Barang*	*Bubaran*	*Pelog*	*Barang*
26.	*Ketawang Ibu Pretiwi Laras Pelog Pathet Nem*	*Ketawang*	*Pelog*	*Nem*
27.	*Ketawang Kinanthi Pawukir Laras Slendro Pathet Manyura*	*Ketawang*	*Slendro*	*Manyura*
28.	*Ketawang Kinanthi Sandhung Laras Slendro Pathet Manyura*	*Ketawang*	*Slendro*	*Manyura*
29.	*Ketawang Langen Gita Laras Pelog Pathet Barang*	*Ketawang*	*Pelog*	*Barang*
30.	*Ketawang Subakastawa Laras Slendro Pathet Sanga*	*Ketawang*	*Slendro*	*Sanga*
31.	*Ladrang Kalongking Laras Pelog Nem*	*Ladrang*	*Pelog*	*Nem*
32.	*Ladrang Mugi Rahayu Laras Slendro Manyura*	*Ladrang*	*Slendro*	*Manyura*
33.	*Ladrang Pariwisata Laras Slendro Sanga*	*Ladrang*	*Slendro*	*Sanga*
34.	*Ladrang Santi Mulya Laras Pelog Lima*	*Ladrang*	*Pelog*	*Lima*
35.	*Ladrang Sumyar Laras Pelog Barang*	*Ladrang*	*Pelog*	*Barang*

In Javanese *gamelan*, there are two *karawitan* playing styles: Surakarta and Yogjakarta [2,3]. This Javanese *gamelan* notation dataset is designed for the Surakarta style. Each song in the dataset consists of [2,3]:

• The main melody of a song with the characteristics of the song, which is used as a guide to play some of the gamelan instruments.
• The notation for various groups of instruments consists of:
  1. Melodic instruments that have a role in playing the melody of the song, are referred to as balungan instruments. Instruments that fall into this category are: demung, saron, and slenthem.
  2. The peking instrument is part of the balungan group, but it differs from other balungan instruments because of its special pattern.
  3. The instruments of bonang barung and bonang penerus are used to decorate the songs to enhance their beauty.
  4. Structural instruments consisting of kenong, kethuk, kempyang, kempul and gong, used for song structure patterns. There are two types of gongs: gong suwuk and gong ageng.

**Fig. 3:**
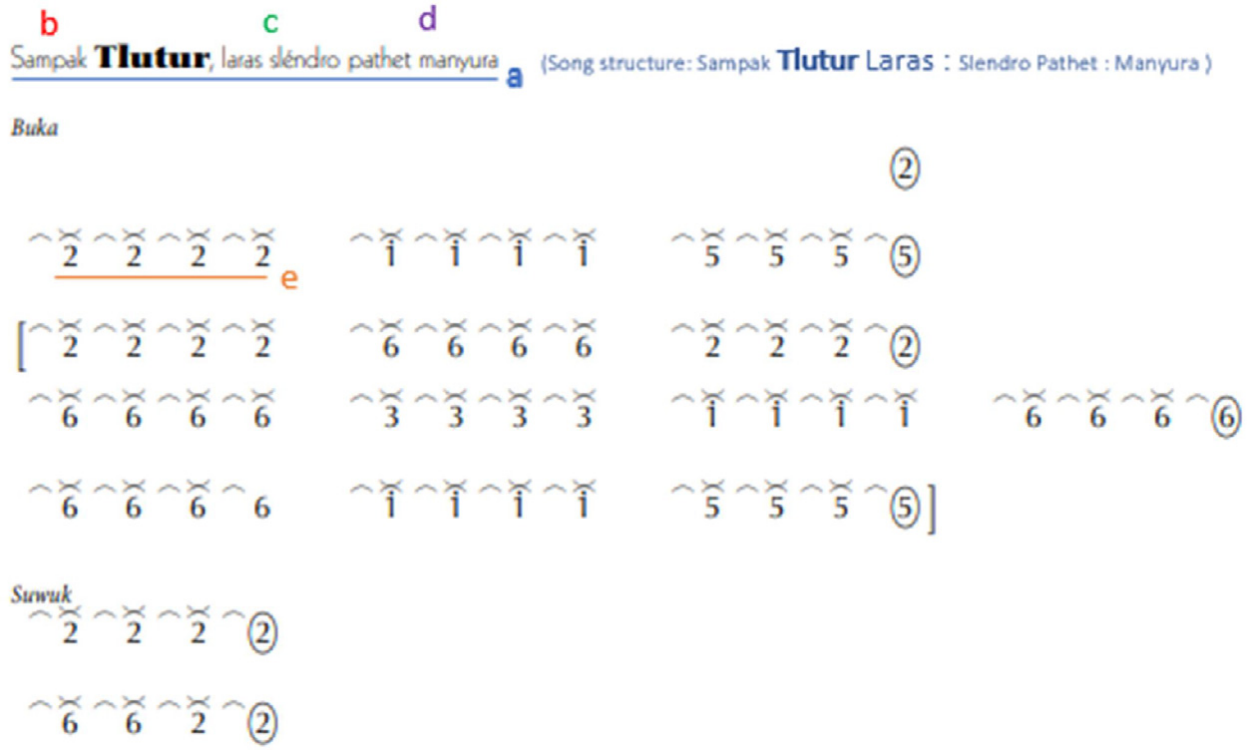
Song of *Sampak Tlutur Laras Slendro Pathet Manyura* (a) title of song (b) type of song structure (c) type of *Laras* (d) type of *Pathet* (e) *gatra.*

The main melody of a song in Javanese *gamelan*, as illustrated in Fig. 3, the song of *Sampak Tlutur Laras Slendro Pathet Manyura* consists of:

(a) Song title which consists of the type of song structure, song name, *laras* and *pathet*.
(b) Song structure determines the pattern of structural instruments.
(c) *Laras* is a scale. *Laras* of Javanese *gamelan* consists of the *pelog* and *slendro.* For one octave of *slendro*, there are five tones, while one octave of *pelog* comprises seven tones. The slendro consists of 1, 2, 3, 5, and 6. However, the *Pelog* consists of 1, 2, 3, 4, 5, 6, and 7.
(d) *Pathet* denotes the atmosphere or character of a particular song. The presentation of *pathet* in Javanese *gamelan* instruments creates a theatrical narrative, as seen in the transition from *pathet nem* to *pathet manyura* in puppet performance, which is used to create an increasingly ascending narrative.

*Laras slendro* is divided into:

1. *Laras Slendro Pathet Nem* employs notes 2 and 5 frequently.
2. *Laras Slendro Pathet Sanga* is characterized by the use of 5 and 1, commonly known as low notes.
3. *Laras Slendro Pathet Manyura* is characterized by its frequent use of high notes, specifically the 6 and 2.

*Laras Pelog* is divided into:

1. *Laras Pelog Pathet Lima* is characterized by descending notes 5, 4, 2, and 1 and is typically used in melancholic songs.
2. *Laras Pelog Pathet Nem* marked by the notes 2, 1, 6, 5. Usually used for powerful songs, such as war scenes.
3. *Laras Pelog Pathet Barang* is identified by the presence of the note 7.
(e) *Gatra* refers to a little piece of a song that contains four melodic notes.

The song sections in Javanese *gamelan* include: *Buka, Ompak, Ngelik,* and *Suwuk. Buka* is the opening or introductory part of the song, *buka* is played by the *bonang barung* instrument. *Ompak* functions as the transition part from the *buka* to the main song (*Ngelik*). *Ngelik* contains the song's lyrics, chorus, or refrain. The *Ompak* and *Ngelik* sections contain notations that are the main melody of the song. The *suwuk* part is played only in the last part of the song. However, it should be noted that not all songs are composed of the four parts.

In the song section, except for *buka*, contains the main melody of a song and symbols of structural instruments for this song as described in Table 2. And the location of these symbols according to the type of song structure.

**Table 2:** Symbols of structural instruments.

No	Instrument	Simbol
1	*Gong Ageng*	circle
2	*Gong Suwuk*	smiley and frowning faces
3	*Kempul*	smiley face
4	*Kenong*	frowning face
5	*Kethuk*	plus sign
6	*Kempyang*	minus sign

Generally, each instrument notation consists of two parts: the melody notation, which is the basic notation of the song, and the instruments notation. However, some data may only present a single part, which means that it shows this instrument's notation.

**Fig. 4:**
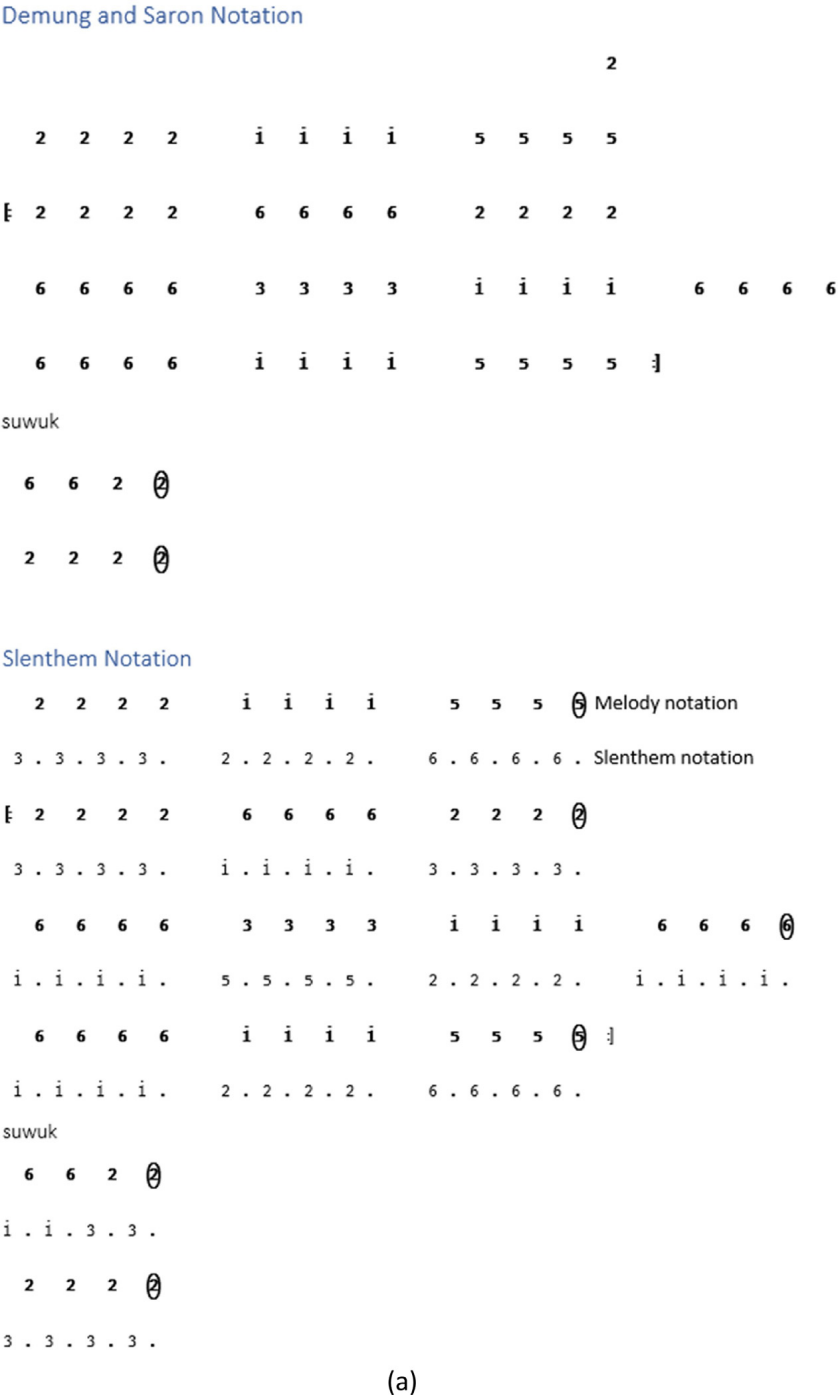

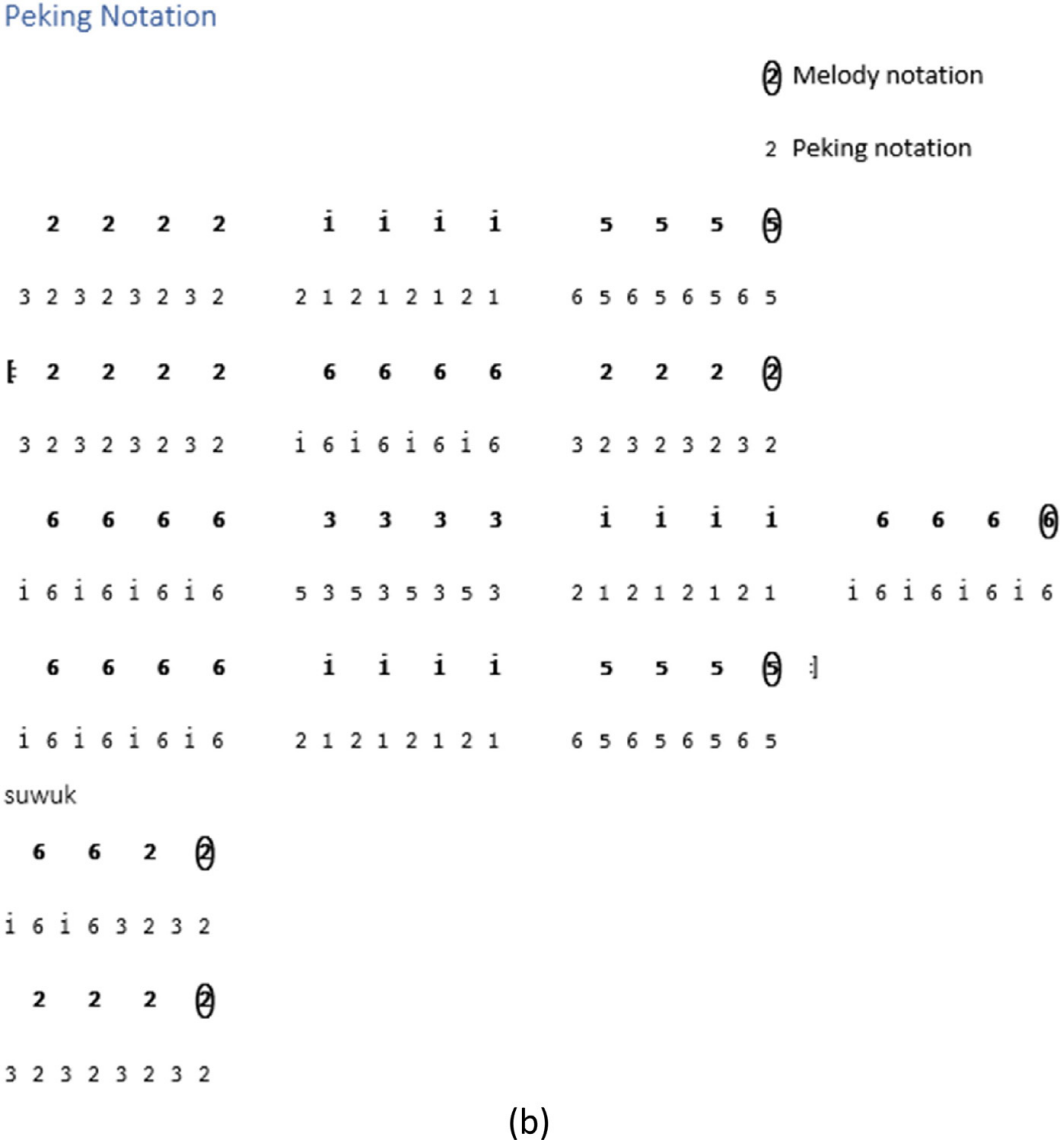

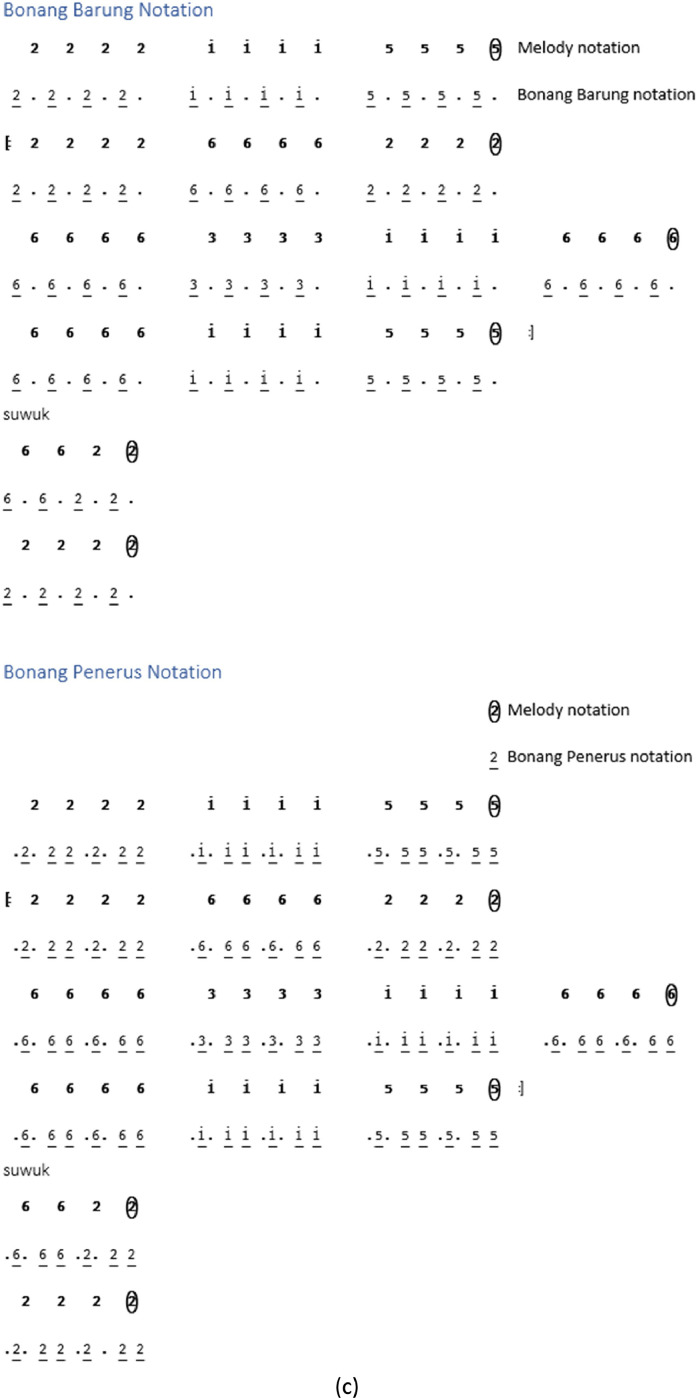

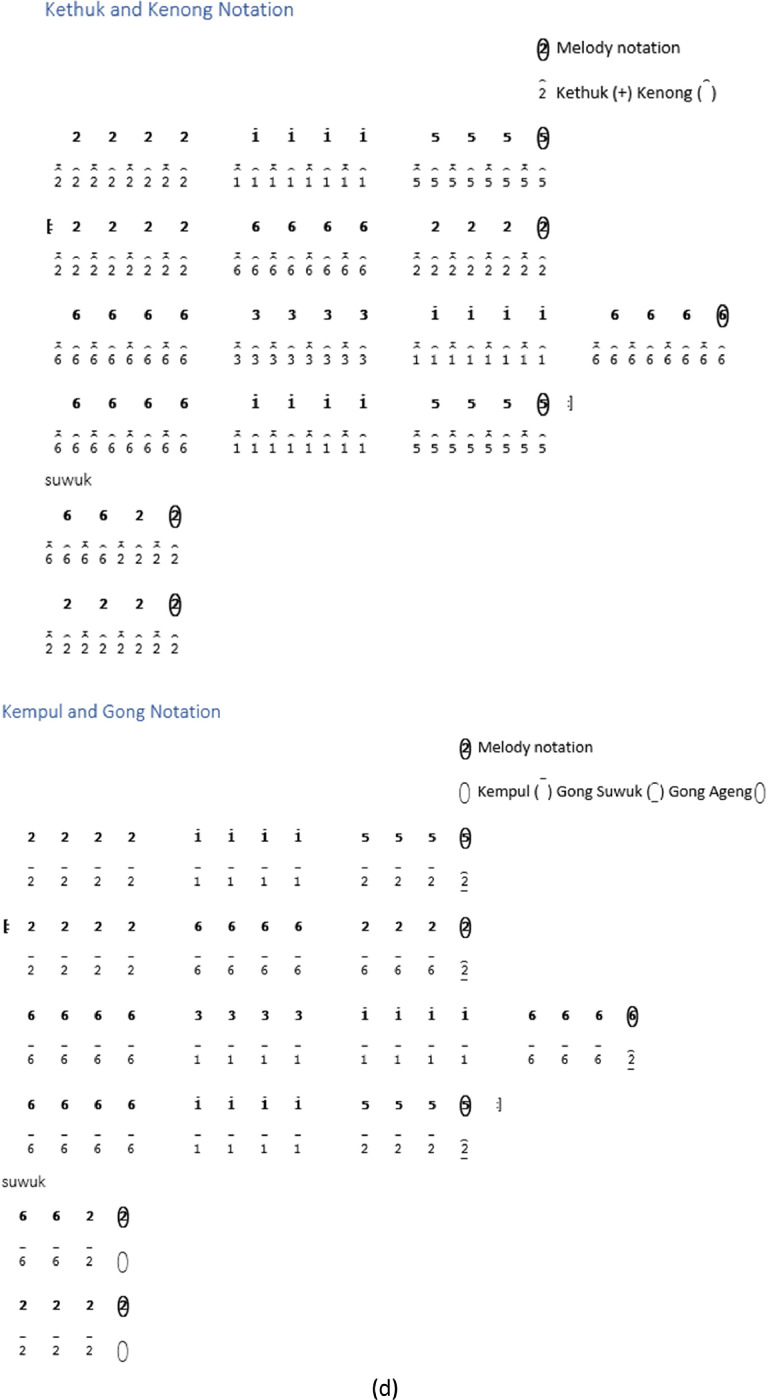
Instruments notation of *Sampak Tlutur Laras Slendro Pathet Manyura* of (a) *Balungan* instrument group, (b) *Peking*, (c) *Bonang Barung* and *Bonang Penerus* instruments, and (d) Structural Instrument Group.

While some instrument notations begin at the *Buka* section, others are directly in the song section. Not all types of song structures have notations for *kempyang* instrument. Only *ketawang* and *ladrang* types include *kempyang* instrument. Therefore, in the case of the song *Sampak Tlutur Laras Slendro Pathet Manyura* in Fig. 4, there is no *kempyang* instrument notation.

## 4 Experimental Design, Materials and Methods

**Fig. 5:**
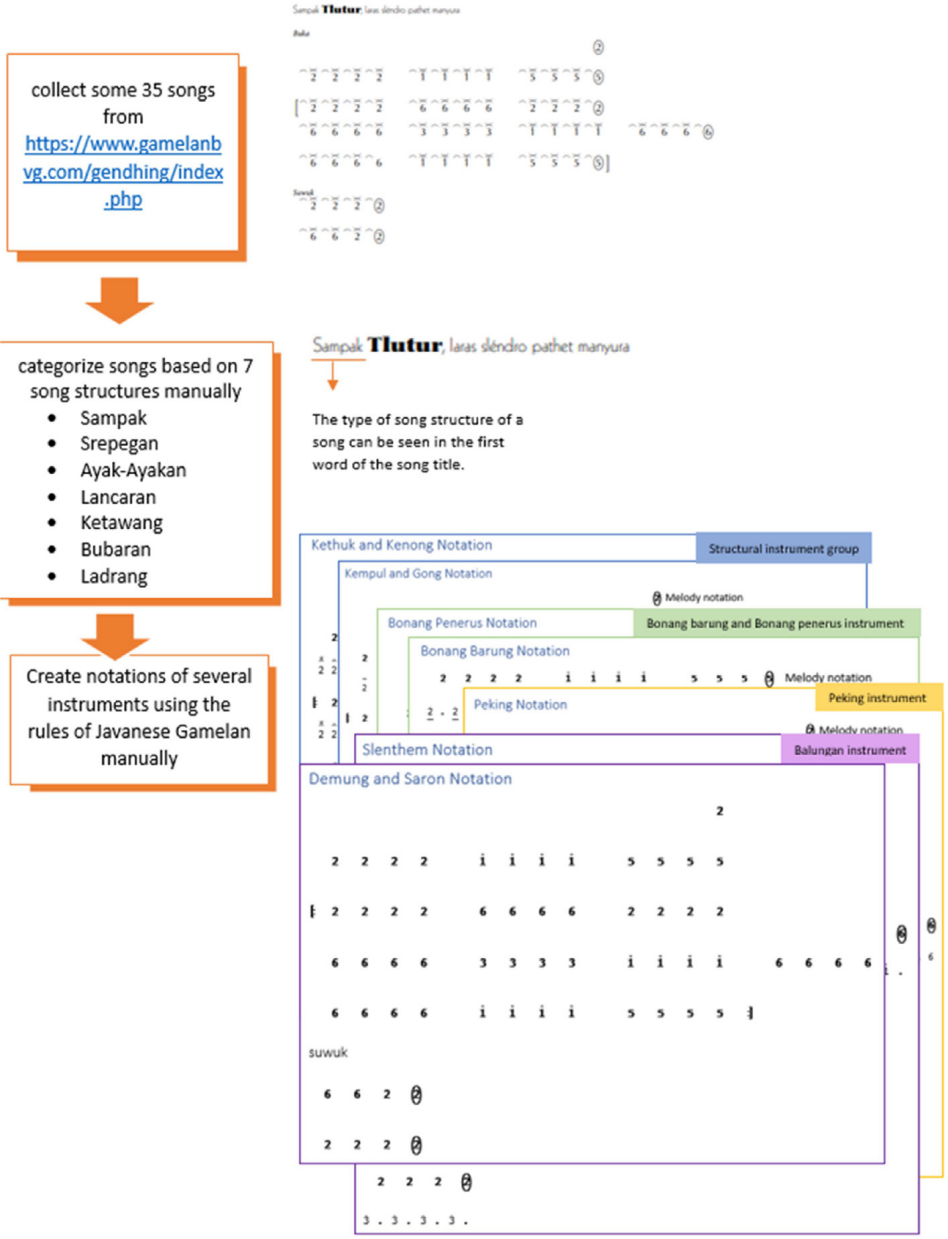
Steps for producing a Javanese *gamelan* notation dataset.

The process of creating the Javanese *gamelan* notation dataset is presented in Fig. 5, which begins with collecting several songs from https://www.gamelanbvg.com/gendhing/index.php (a total of 35 songs), then grouping them based on song structure, and finally completing all the notations of each *gamelan* instrument group based on the rules of each instrument. This notation process was executed manually and the results were saved in PDF format with *gamelan* symbol notation.

A Javanese *gamelan* song typically consists of the primary melody and some additional information to indicate how the song should be played. This is not an issue for experienced *gamelan* musicians. They can utilize the song's instructions to connect the rules of playing other instruments *gamelan*. For novice *gamelan* players, it may be difficult because in Javanese *gamelan*, the playing pattern of each instrument has a variety of different variations depending on several factors such as: melody notation patterns on *balungan* instruments, types of song structures, rhythms used, scales, song modes, and *nada seleh*. To solve this problem, this dataset provides complete notation information for several instruments.

The following are the rules for determining the *gamelan* playing pattern on each instrument [3]:

1. The notation guidelines for the *balungan* instrument group, which includes the *demung, saron,* and *slenthem* instruments, are as follows:
• *Demung, saron,* and *slenthem* instruments are noted in accordance with the primary melody notation.
• With the exception of the *Sampak* song structure type, the *slenthem* instrument is noted one level above the melody note, as demonstrated in Fig. 6.

**Fig. 6:**
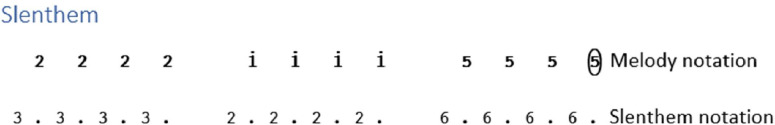
Example rule of *slenthem* instrument implementation in the *Sampak Tlutur Slendro Pathet Manyura* song.
2. Notation rules for instrument groups are influenced by various factors, including the *peking* instrument and the *bonang barung* and *bonang penerus* instrument groups. The *peking* instrument notation is affected by the rhythm and melody notation pattern, whereas for the *bonang barung* and *bonang penerus* groups, it is also influenced by *Laras* and *Pathet* information and *Nada Seleh*.
(a) Rhythm

The details of the rhythm of the song are mostly unwritten, because the rhythm is part of the of the performance. The element that determines the rhythmic pattern is the *kendhang*, but this does not provide any information about the *kendhang* pattern being used. By default, the rhythm of a song is determined by the type of song structure. Additional information about default rhythm patterns for each song structure can be found in Table 3.

(b) The melody notation pattern

**Table 3:** List rhythms according to the type of song structure.

No	Song structure	The rhythm used
1	*Sampak*	*Irama Tanggung*
2	*Srepegan*	*Irama Tanggung*
3	*Ayak-ayakan*	*Irama Tanggung* then moves to *Irama dadi*
4	*Lancaran*	*Irama Lancar*
5	*Ladrang*	*Irama Tanggung*
6	*Ketawang*	*Irama Tanggung* then moves to *Irama dadi.*
7	*Bubaran*	*Irama Tanggung*

The notation patterns of *bonang barung* and *bonang penerus* instruments are influenced by the melody notation patterns performed by the *balungan* instrument in a song. Here are a few examples of *balungan* notation patterns:

1. *Balungan Nibani* involves melodic notes within a *gatra*, where odd-numbered notes are either left empty or marked with a dot.
2. *Balungan Mlaku* is the arrangement of melodic notes in a gatra, where all the tones are filled.
3. *Balungan Gantung* is characterized by a gatra containing a pair of twin tones on the first and second or third and fourth notes.
(c) *Nada Seleh* represents the concluding note in the *balungan* notation for each line of a song or the last note of the piece of song. Table 4 presents the *Sekaran* pattern for *Bonang Barung* and *Bonang Penerus*, focusing on the *Nada Seleh* in each line of the song. In contrast, Table 5 represents the *Imbal* pattern for the same instruments, but with attention to the last note of the song.

**Table 4:** The *Sekaran* pattern of *Bonang Barung* and *Bonang Penerus* instrumens.

*Nada Seleh* of each line	Pattern for *Bonang Barung*	Pattern for *Bonang Penerus*
1	1 1 1 . 1 1 1 1	2.2.2.2. 2.2.2.2.
2	5612 2161612 atau 6356 56532	51561612 22161612
3	3 3 3 . 3561653 atau 31 5 31 . 31561653	5.5.5.5. 61216523
5	5 5 5 . 3561615 atau 5612 2161615	51561612 22161615
6	1666 3561216	51356123 51535156

**Table 5:** The *Imbal* pattern of *Bonang Barung* and *Bonang Penerus* instruments.

*Laras* and *Pathet*	*Nada Seleh* of the last song	Pattern for	
		*Bonang Barung*	*Bonang Penerus*
*Laras Slendro Pathet Manyura*	1,2,3,5	1-3	2-5
	6	3-6	5-1/5-2
*Laras Pelog Pathet Barang*	7,2,3,5	7-3	2-5
	6	3-6	5-7/5-2
Others	1,2,3,5	6-2	1-3
	6	3-6	5-2/5-7

In Pelog Barang: Notation 1 replaced by 7

### 4.1 *Peking* instrument rule

*Peking* instrument is actually a group of *balungan* instruments, but *peking* instrument has different techniques from other *balungan* instruments. There are various variations in *peking* instruments by duplicating the melody note, but sometimes *gamelan* experts make it more varied by using an alternating model by changing positions.

**Fig. 7:**
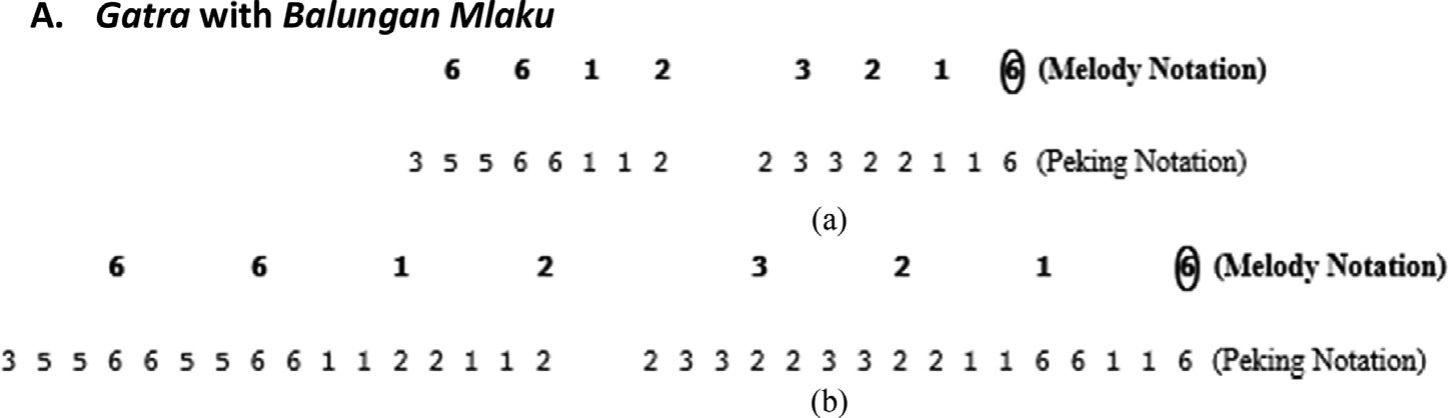
Example rule of the implementation *peking* instrument in *Balungan Mlaku* with (a) *Irama Tanggung* (b) *Irama Dadi* in the *Ladrang Kalongking Laras Pelog Pathet Nem* song.
A. ***Gatra* with *Balungan Mlaku***

I. For all song structures except *Sampak*, a variation pattern is used where each *balungan* note is repeated twice and then shifted to the next *gatra*. For details, see Fig. 7, which shows the notation on the *peking* instrument with *Irama Tanggung* and *Irama Dadi*.

Explanation of Fig. 7a: *Balungan* notation 6612 transforms into 66,66,11,22, which then becomes 55,66,11,22 (in order to add variety, the twin tone 6 is replaced with 5). The sequence then transforms into 35,56,61,12 (This change is due to the initial tone (3) being shifted from the previous *gatra*, so there is an additional note 3, and the final note 2 is shifted to the next *gatra*).

Explanation of Fig. 7b: The *peking* notation in *Irama Tanggung* is duplicated, and the last note of each *gatra* is moved to the next *gatra*, thereby duplicating the data as shown in Fig. 7b.

B. ***Gatra* with *Balungan Nibani***

**Fig. 8:**
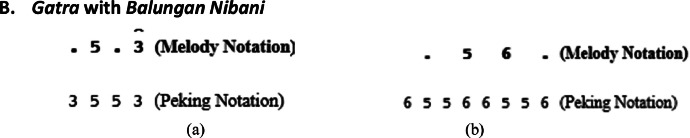
Example rule of the implementation peking instrument in Balungan Nibani (a) Irama Lancar in the Lancaran Manyar Sewu Laras Slendro Pathet Manyura song (b) Irama Tanggung in the Ladrang Kalongking Laras Pelog Pathet Nem song.

Explanation of Fig. 8a: If the *gatra* pattern of the *balungan nibani* shown in Fig. 8a is found, then the *peking* pattern becomes 5 5 3 3 and then become 3 5 5 3. The first note 3 is obtained from the previous *gatra*, and then the last note 3 is shifted to the next *gatra*.

Explanation of Fig. 8b: If the *gatra* pattern of *balungan nibani* is as shown in Fig. 8b, then the transformation for the *balungan* note must be modified to . 5 . 6, so that the *peking* note becomes 5 5 6 6 5 5 6 6 and then 6 5 5 6 6 5 5 6, the first note 6 is taken from the previous *gatra*, and the last note 6 is shifted to the next *gatra*.

C. ***Gatra* with *Balungan Gantung***

**Fig. 9:**
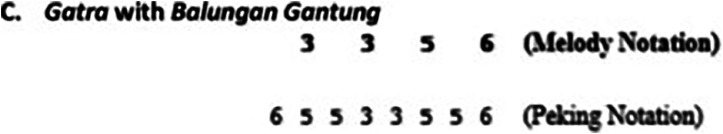
Example rule of the implementation *peking* instrument in *Balungan Gantung* with *Irama Tanggung* in the *Ladrang Kalongking Laras Pelog Pathet Nem* song.

If a *gatra* pattern with a twin-note pattern is identified, as shown in Fig. 9 (i.e., note 3), then the transformation for the *balungan* note is changed to 5 3 5 6 or 2 3 5 6 (by taking the upper or lower note). Subsequently, the *peking* pattern will become 5533 5566 or 2233 5566. If the 5533 5566 is chosen and then the first note 6 obtained from the previous *gatra*, and the last note 6 shifted to the subsequent *gatra*, resulting in the final *peking* notation being 6553 3556.

**Fig. 10:**
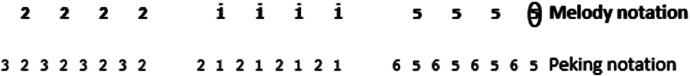
Example rule of the implementation *peking* instrument in the *Sampak Tlutur Laras Slendro Pathet Manyura* song.

II. In *Sampak*, the *peking* instrument notation follows an alternating doubling pattern, shown in Fig. 10. The melody contains twin tones (2222), therefore the *peking* notation takes the upper note (3232), because the rhythm used in this song is *Irama Tanggung*, so the *peking* notation becomes 3232 3232.

### 4.2 Bonang Barung and Bonang Penerus instruments rule

The *bonang barung* and *bonang penerus* instrument groups have various playing patterns, including *Gembyangan, Mipil*, a combination of *Gembyangan* and *Mipil*, and *Imbal Sekaran*. The playing pattern of *bonang penerus* is to follow the *bonang barung*. Therefore, the initial step is to select patterns for *bonang barung* followed by *bonang penerus*.

**Fig. 11:**
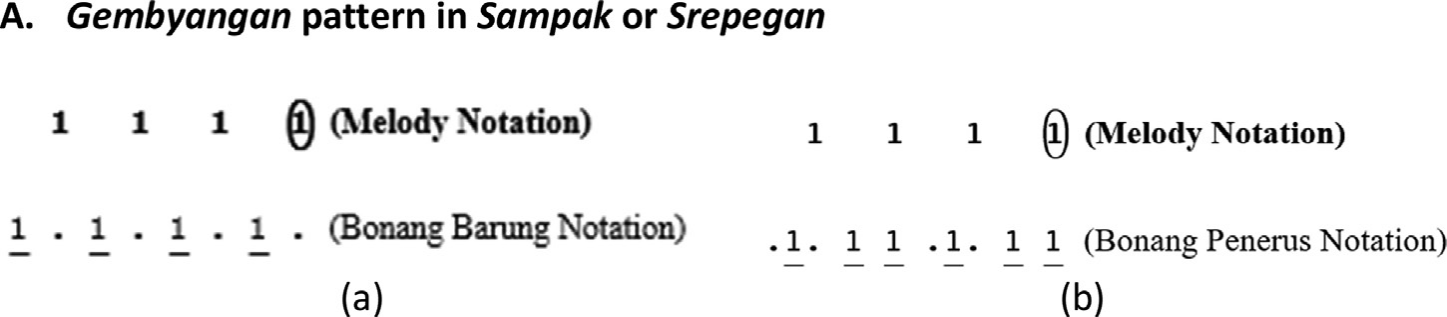
Example rule of the implementation Gembyangan pattern with Irama Tanggung for (a) Bonang Barung (b) Bonang Penerus in the Sampak Mayura Laras Slendro Pathet Manyura song.

**Fig. 12:**
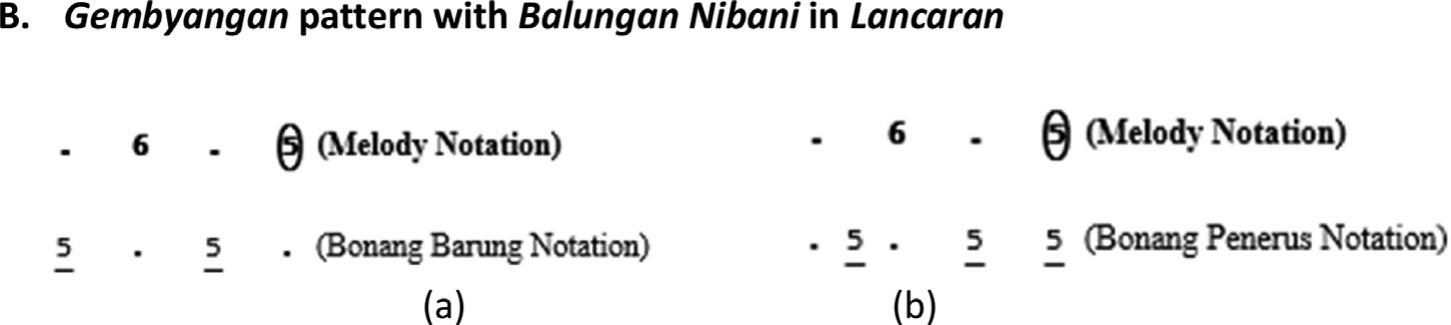
Example rule of the implementation Gembyangan pattern with Irama Lancar for (a) Bonang Barung (b) Bonang Penerus in the Lancaran Manyar Sewu Laras Slendro Pathet Manyura song.

**Fig. 13:**
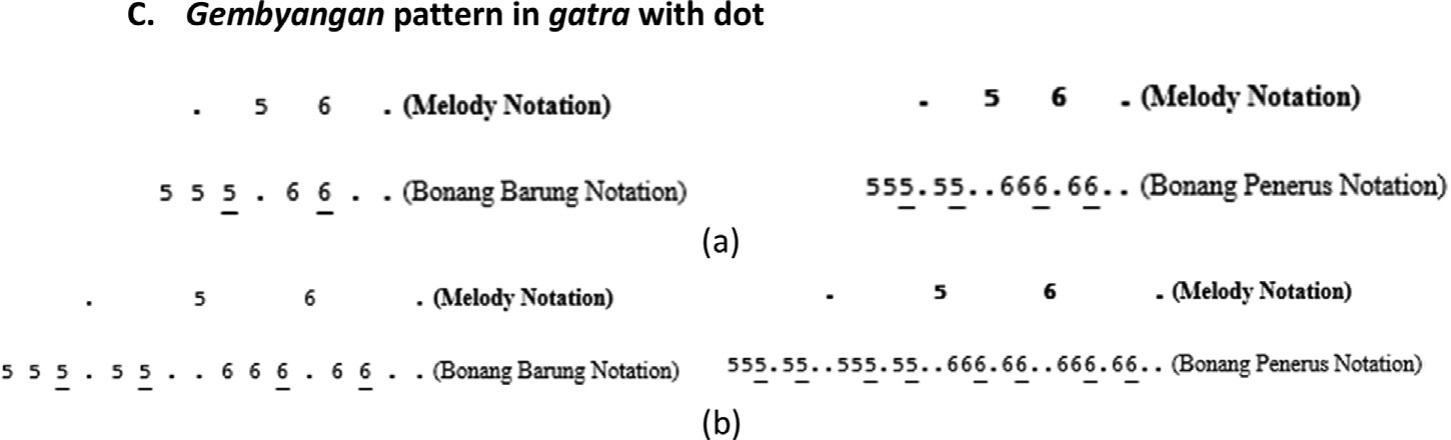
Example rule of the implementation *Gembyangan* pattern with a melody that has a dot for *Bonang Barung* and *Bonang Penerus* with (a) *Irama Tanggung* (b) *Irama Dadi* in the *Ladrang Kalongking Laras Pelog Pathet Nem* song.

**Fig. 14:**
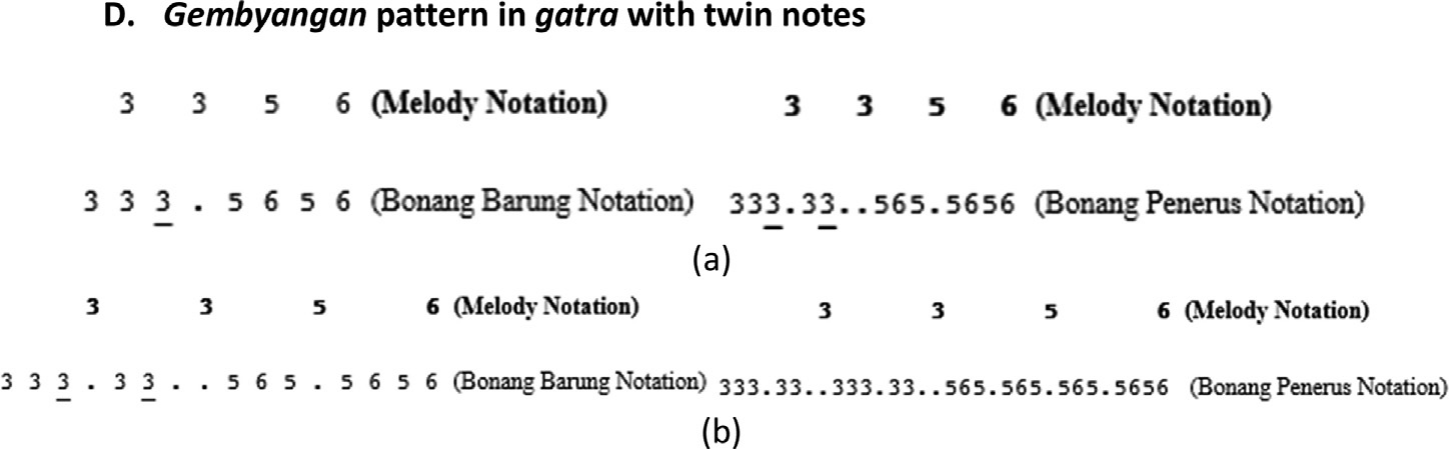
Example rule of the implementation *Gembyangan* pattern with melodies with twin notes for *Bonang Barung* and *Bonang Penerus* with (a) *Irama Tanggung* (b) *Irama Dadi* in the *Ladrang Kalongking Laras Pelog Pathet Nem* song.
A. ***Gembyangan* pattern in *Sampak* or *Srepegan***
B. ***Gembyangan* pattern with *Balungan Nibani* in *Lancaran***
C. ***Gembyangan* pattern in *gatra* with dot**
D. ***Gembyangan* pattern in *gatra* with twin notes**

I. *Pola Gembyangan,* which is the playing of two notes *(i.e.,* a lower note and an upper note at the same time), is written as 2, which means the playing of a note of 2 and a note of 2͘. The *gembyangan* pattern is often used in *Sampak, Srepegan* and *Lancaran*, and special conditions, i.e., *gatra* with dot or twin notes (first and second or third and fourth notes). Some examples of the use of the *Gembyangan* pattern are shown in Fig. 11, Fig. 12, Fig. 13, Fig. 14:

Therefore, the variation of this *gembyangan* pattern depends on the type of song structure, melody notation pattern, and rhythm used.

**Fig. 15:**
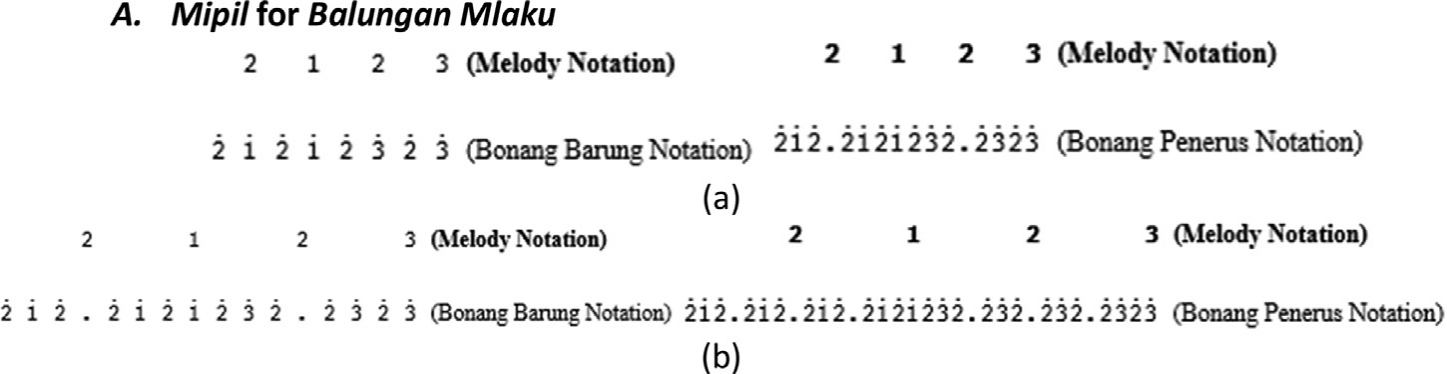
Example rule of the implementation Mipil pattern with Balungan Mlaku for Bonang Barung and Bonang Penerus with (a) Irama Tanggung (b) Irama Dadi in the Ladrang Kalongking Laras Pelog Pathet Nem song.

**Fig. 16:**
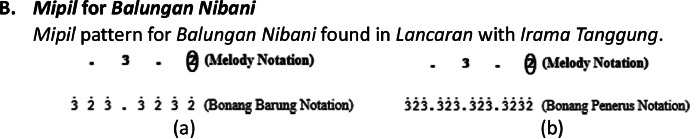
Example rule of the implementation Mipil pattern with Balungan Nibani for (a) Bonang Barung and (b) Bonang Penerus with Irama Tanggung in the Lancaran Maesa Kurda Laras Slendro Pathet Sanga song.

**Fig. 17:**
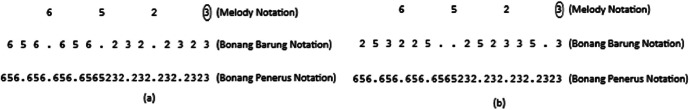
Example rule of the implementation *Mipil* pattern for melody with low pitch for *Bonang Barung* and *Bonang Penerus* with *Irama Dadi* using (a) general pattern (b) varied pattern in the *Ketawang Langen Gita Laras Pelog Pathet Barang* song.
A. ***Mipil* for *Balungan Mlaku***

II. *Mipil* pattern, which is playing each note of the *bonang barung* one by one by using 2 notes from the *balungan. Mipil* is used for *Irama Tanggung* and *Irama Dadi*, the *mipil* pattern also depends on the type of melody pattern on the *balungan*, as shown in Fig. 15, Fig. 16, Fig. 17.

In the *Mipil* pattern for the *Irama Dadi*, the transformation was originally 2͘1͘͘2͘1͘͘2͘1͘͘2͘1͘͘2͘3͘2͘3͘2͘3͘2͘3͘ on the *bonang barung*, to keep it interesting, the transformation of the third note is replaced by a dot, making it 2͘1͘͘2͘.2͘1͘͘2͘1͘͘2͘3͘2͘.2͘3͘2͘3͘, as shown in Fig. 15b.

B. ***Mipil* for *Balungan Nibani***

*Mipil* pattern for *Balungan Nibani* found in *Lancaran* with *Irama Tanggung*.

C. ***Mipil* with low-pitched in *Irama Dadi***

**Fig. 18:**
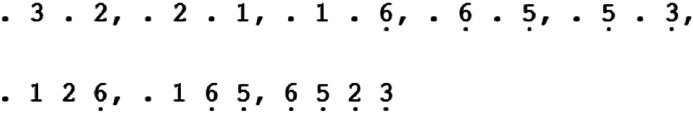
Melody of *balungan* with low pitch.

*Mipil* pattern on *balungan nibani* or *mlaku* for *Irama Dadi* with the low pitch. Actually, the pattern of *bonang barung* and *bonang penerus* in this pattern is the same as in the *Irama Tanggung* (Fig. 17a), but often *gamelan* experts make creations that change the notation pattern on the *bonang barung* instrument, as shown in Fig. 17b.
The *bonang barung* pattern is often used for low-pitched in *balungan nibani* or *mlaku*, as shown in Fig. 18.

III. Combine *Gembyangan* and *Mipil*

In general, *bonang barung* notation patterns commonly use a combination of *gembyangan* and *mipil*. Specifically, *Gembyangan* is used when there is a dot on a pair of notes (first and second, or third and fourth) in a *gatra*, and *Mipil* is used when there is no dot on the other pair. For instance, in a *gatra* with a melody note of .132, then on note .1 use *gembyangan*, then on note 32 use *mipil* in the notation of *bonang barung* and *bonang penerus*.

IV. *Imbal Sekaran*

**Fig. 19:**
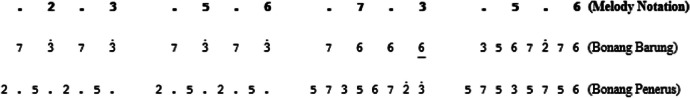
Example rule of the implementation *Imbal Sekaran* pattern in the *Lancaran Kuda Nyongklang Laras Pelog Pathet Barang* song.

The *Imbal Sekaran* patterns on the *bonang barung* and *bonang penerus* often accompany *gamelan* singers in the *Lancaran* with *Irama Lancar*. In addition to the type of *laras* and *pathet* of the song, *Nada seleh* is also a crucial factor for defining the pattern of *bonang barung* and *bonang penerus*.
In Fig. 19, the *Lancaran Kuda Nyongklang Pelog Barang* song is presented with the *Imbal Sekaran* pattern with a reference nada seleh 6 within the song line for the *Sekaran* pattern reference in the third and fourth *gatra*, and a reference nada seleh 7 at the end of the song for the *Imbal* pattern reference in the first *gatra* and second *gatra*.

### 4.3 Structural instruments rule

Notation rules for the structural instrument group. In this group, the notation pattern is influenced by the song structure. The structural instrument groups are *Kethuk, Kenong, Kempyang, Kempul,* and *Gong* (*Gong Suwuk* and *Gong Ageng*). *Kethuk and Kempyang* are single-pitched instruments. The following is the notation pattern of structural instruments based on the type of song structure.

I. *Sampak*

**Fig. 20:**
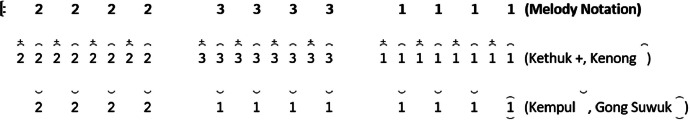
Example rule of the implementation Structural instruments in the *Sampak Manyura Laras Slendro Pathet Manyura* song.

The *Sampak* song structure consists of 2, 3, and 4 *gatras* in each gong. Fig. 20 provides an example of the implementation of structural groups in *Sampak*. The pattern rules for each instrument are as follows:

- *Kenong* is played twice (half tempo of *balungan*) using the *balungan* note.
- *Kethuk* is played before each *balungan* note.
- *Kempul* is generally played according to the *balungan* note. However, occasionally, it can use different notes than the *balungan*.
- *Gong suwuk* is played at the end of each line of notation.
- *Gong ageng* is played in each line of the *Suwuk* section.
II. *Srepegan*

**Fig. 21:**
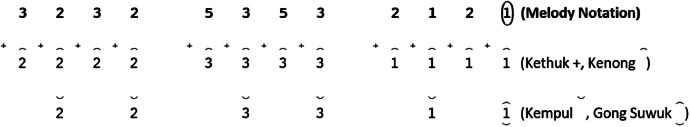
Example rule of the implementation Structural instruments in the *Srepegan Manyura Laras Slendro Pathet Manyura* song.

In *Srepegan* song structure, the amount of *gatra* in a *gong* is not fixed. Fig. 21 illustrates an example of the implementation of structural groups in *Srepegan*. The pattern rules for each instrument are as follows:

- *Kenong* is played on the *balungan* note, using the last note of each *gatra*.
- *Kethuk* is played before the *balungan* note.
- *Kempul* is played on the even *balungan* note of each *gatra*, using this *balungan* note.
- *Gong suwuk* is played at the end of each line of notation.
- *Gong ageng* is played in each line of the *Suwuk* section.
III. *Ayak-ayakan*

**Fig. 22:**
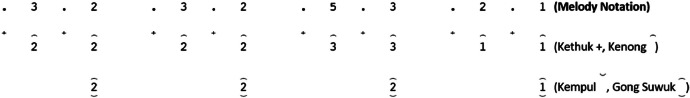
Example rule of the implementation Structural instruments in the *Ayak-ayak Manyura Laras Slendro Pathet Manyura* song.

The *Ayak-ayakan* song structure has an irregular number of *gatras* in a *gong*. For an example of the implementation of structural groups in *Ayak-ayakan*, see Fig. 22. The pattern rules for each instrument are as follows:

- *Kenong* is played on the even *balungan* note of each *gatra*, using this *balungan* note.
- *Kethuk* is played before *Kenong*.
- *Gong suwuk* is played at the end of each line of notation.
- *Gong ageng* is located at the end of the song.
IV. *Lancaran*

**Fig. 23:**
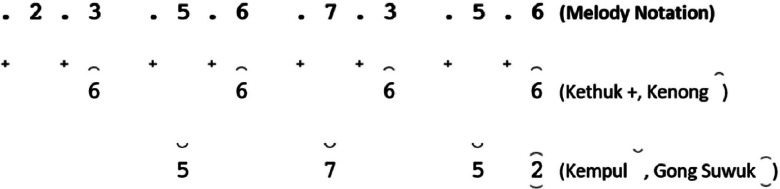
Example rule of the implementation Structural instruments in the *Lancaran Kuda Nyongklang Laras Pelog Pathet Barang* song.

The *Lancaran* song structure consists of four *gatras* in a single *gong*. Fig. 23 presents an example of the implementation of structural groups in *Lancaran*. The pattern rules for each instrument are as follows:

- *Kenong* is located at the end of each *gatra*, using the last note of the *balungan* in the even *gatra*.
- *Kethuk* is placed on the odd *balungan* note of each gatra.
- *Kempul* is placed on the second note of each *gatra*, except the first *gatra* is left blank, using this *balungan* note.
- *Gong suwuk* is played at the end of each line of notation.
- *Gong ageng* is played at the end of each song section.
V. *Bubaran*

**Fig. 24:**
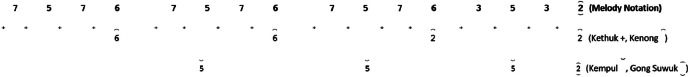
Example rule of the implementation Structural instruments in the *Bubaran Arum Arum Laras Pelog Pathet Barang* song.

The *Bubaran* has a similar structure to *Lancaran*. Fig. 24 presents an illustration of how structural groups are implemented in *Bubaran*. The pattern rules for each instrument are as follows:

- *Kenong* is located at the end of each *gatra*, using the last note of the balungan in the even *gatra*.
- *Kethuk* is played before the *balungan* note.
- *Kempul* is placed on the second note of each *gatra*, except the first *gatra* is left blank, using this *balungan* note.
- *Gong suwuk* is played at the end of each line of notation.
- *Gong ageng* is located at the end of the song.
VI. *Ketawang*

**Fig. 25:**
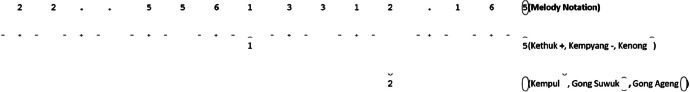
Example rule of the implementation Structural instruments in the *Ketawang Ibu Pertiwi Laras Pelog Pathet Nem* song.

The *Ketawang* song structure has 4 *gatras* in a single gong. An example of the implementation of structural groups in *Ketawang* can be seen in Fig. 25. The pattern rules for each instrument are as follows:

- *Kenong* is located on the last note of the 2nd and 4th *gatra*, using this *balungan* note.
- *Kethuk* is placed on the first and third *balungan* note of each gatra.
- *Kempyang* is played before and after *Kethuk*.
- *Kempul* is placed on the last note of the 3rd *gatra*, using this *balungan* note.
- *Gong ageng* is played at the end of each line of notation.
VII. *Ladrang*

**Fig. 26:**
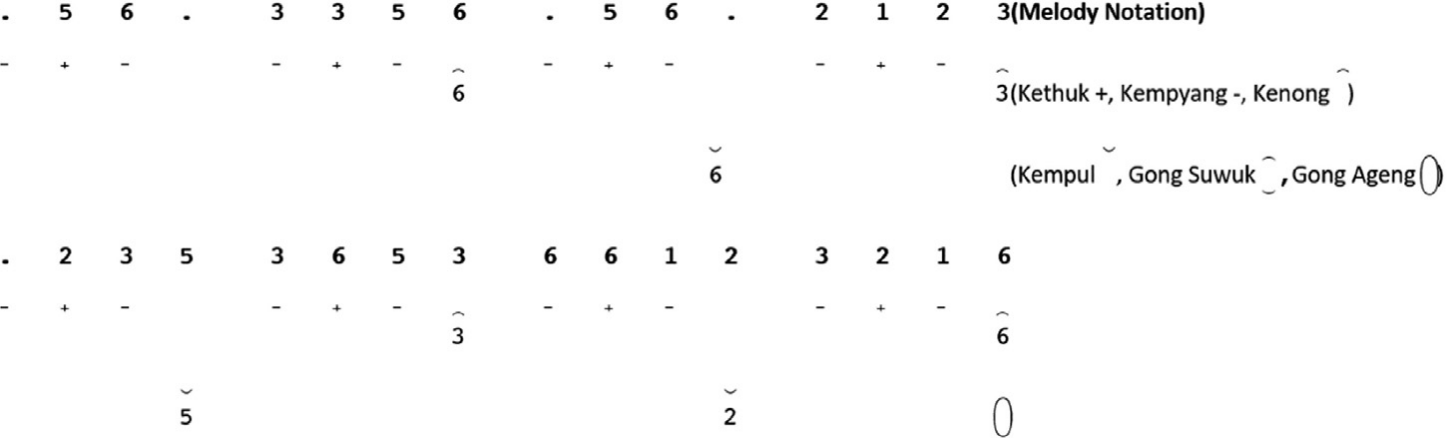
Example rule of the implementation Structural instruments in the *Ladrang Kalongking Laras Pelog Pathet Nem* song.

The structure of a *Ladrang* comprises of 8 *gatras* within a single *gong*. Fig. 26 illustrates an example of the implementation of structural groups in *Ladrang*. The pattern rules for each instrument are as follows:

- *Kenong* is *located* on the last note of each even *gatra*, using this *balungan* note.
- *Kethuk* is placed on the second *balungan* note of each gatra.
- *Kempyang* is played on the even *balungan* note of each gatra.
- *Kempul* is placed on the last note of the 3rd *gatra*, using this *balungan* note.
- *Gong ageng* is played at the end of each line of notation.

## Limitations

This dataset only contains a limited number of Javanese *gamelan* song structures, which are categorized as short songs. Unfortunately, more complex song structures, such as *Gerongan, Gendhing, Jineman, Langgam*, and others, are not included in this particular dataset. Additionally, the *Kendhang* instrument is not available for analysis.

## Ethics Statement

The current work does not involve human subjects, animal experiments, or any data collected from social media platforms.

## CRediT Author Statement

**Arik Kurniawati**: Conceptualization, Methodology, and original draft preparation. **Eko Mulyanto Yuniarno**: Technical Validation, Visualization, and Reviewing. **Yoyon Kusnendar Suprapto**: Reviewing and Supervision. **Noor Ifada**: Writing and Reviewing. **Nur Ikhsan Soewidiatmaka:** Data Validation**.**

## Data Availability

Javanese Gamelan Notation (Original data) (Mendeley Data).
